# Fluid Retention over the Menstrual Cycle: 1-Year Data from the Prospective Ovulation Cohort

**DOI:** 10.1155/2011/138451

**Published:** 2011-08-08

**Authors:** Colin P. White, Christine L. Hitchcock, Yvette M. Vigna, Jerilynn C. Prior

**Affiliations:** ^1^McMaster University Medical Centre, 1280 Main Street West, Hamilton, ON, Canada L8S 4L8; ^2^Centre for Menstrual Cycle and Ovulation Research, Division of Endocrinology, Department of Medicine, University of British Columbia and Vancouver Coastal Health Research Institute, 2775 Laurel Street, 4th Floor, Vancouver, BC, Canada V5Z 1M9

## Abstract

We report menstrual and mid-cycle patterns of self-reported “fluid retention” in 765 menstrual cycles in 62 healthy women. Self-reported “fluid retention,” commonly described as bloating, is one element of the clinical assessment and diagnosis of premenstrual symptoms. These daily diary data were collected as part of an observational prospective one-year study of bone changes in healthy women of differing exercise characteristics. Ovulation was documented by quantitative basal temperature analysis, and serum estradiol and progesterone levels were available from initial and final cycles. Fluid retention scores (on a 0–4 scale) peaked on the first day of menstrual flow (mean ± SE : 0.9 ± 0.1), were lowest during the mid-follicular period, and gradually increased from 0.22 ± 0.05 to 0.50 ± 0.09 over the 11 days surrounding ovulation. Mid-cycle, but not premenstrual, fluid scores tended to be lower in anovulatory cycles (ANOVA *P* = 0.065), and scores were higher around menstruation than at midcycle (*P* < 0.0001). Neither estradiol nor progesterone levels were significantly associated with fluid retention scores. The peak day of average fluid retention was the first day of flow. There were no significant differences in women's self-perceived fluid retention between ovulatory and anovulatory cycles.

## 1. Introduction

Many women perceive changes in fluid retention or “bloating” over the course of their menstrual cycle. As early as 1934, Sweeney [1] reported a pattern of “menstrual edema,” premenstrual weight gain peaking at the onset of flow, in a subgroup of student nurses. Several prospective daily rating studies reported peak fluid retention at the onset of menstrual flow [2, 3], but the hormonal factors underlying these changes remain poorly understood. In particular, it is not clear whether ovulation is necessary, or whether similar changes also occur in anovulatory cycles of normal length.

Although oligomenorrheic menstrual cycles are usually anovulatory, anovulation can also occur in clinically unremarkable menstrual cycles of normal length [4, 5]. In a normally ovulatory menstrual cycle, estradiol has two peaks, the higher mid-cycle peak before ovulation and the luteal peak after ovulation. Progesterone, by contrast, is low during the entire follicular phase but rises following ovulation and remains high during the luteal phase. Both estradiol and progesterone levels are low during the first few days of menstrual flow. During anovulatory cycles, estradiol levels may be variable or tonically high with anovulatory androgen excess (also called polycystic ovary syndrome) or variable but normal [6]. 

Hypothalamic causes for ovulation disturbances include situational stresses [7], cognitive dietary restraint [8, 9], travel, and increases in strenuous exercise with inadequate energy intake [10]. The runners in this study were required to have a well-established running habit with regular menstrual cycle length and to be normally ovulatory before enrollment; running habit was not associated with ovulation disturbances within this group of women [4]. In previous work from this laboratory we have shown that increasing exercise training decreases fluid retention within ovulatory cycles [11].

Assessing ovulation in prospective studies requires compromise between the desire for precise measurement and the need for an accessible, affordable, and acceptable method. Basal temperature methods have fallen out of use because the traditional subjective assessment methods were poorly repeatable [12] and because the rise in basal temperature follows ovulation, and so is not helpful in timing conception. We use quantitative basal temperature (QBT) as a validated, inexpensive way of estimating when and whether ovulation occurred that is compatible with a prospective study. This quantitative algorithm has been validated against the day of peak serum luteinizing hormone (LH) [13] and against ovulatory status by daily urinary PdG [14]. 

This analysis is based on one-year of prospective observational menstrual cycle diary data from premenopausal, initially ovulatory women, who were either normally active or were recreational runners. Following two initially ovulatory screening cycles, many women went on to experience either short luteal phase or anovulatory cycles during the year. Our initial hypotheses were (1) fluid retention would be maximal during the premenstrual days and decrease thereafter, (2) fluid retention would be greater in anovulatory than in ovulatory cycles given the absence of progesterone's potential antimineralocorticoid action, and (3) fluid retention would be lower in exercising women than in those who were normally active.

## 2. Material and Methods

The primary outcome of the study and the study design have been published [4]. Briefly, this was a one-year prospective observational study of normally active women and two groups of runners, regular recreational runners and those who were planning to run a marathon during the study year. Women were asked to keep a record of daily experiences using a structured semiquantitative daily menstrual cycle diary [15, 16]. The daily diary component of the study was unfunded, but our intention has always been to use these data to understand and describe patterns in women's experience during the menstrual cycle in a population of women who are not seeking clinical care, and whose menstrual cycles are clinically unremarkable. This study was conducted from 1985–87 in Vancouver, British Columbia, Canada. The Clinical Screening Committee for Research Involving Human Subjects of the University of British Columbia approved the study. All women were volunteers, and gave written informed consent.

### 2.1. Participants

Healthy, nonsmoking, premenopausal women were recruited from the community, and were screened by questionnaire and prospective collection of daily basal temperature and menstrual cycle diary records in two complete menstrual cycles. The initial questionnaire exclusion criteria were ages <20 or > 42, hormonal contraceptive or bone active drug use (e.g., glucocorticoids) within six months, body mass index <17 or > 27, weight change of more than 2.5 kg within the past year, smoking, shift work, clinical or biochemical androgen excess, and eating disorders or compulsive exercising. Participants completed a baseline questionnaire of reproductive history and demographic variables. Of the initial 245 women, 113 met questionnaire criteria, and, of those, 81 met ovulation criteria for two consecutive normally ovulatory cycles (cycle length 21–36 days, luteal length ≥ 10 days by quantitative basal temperature [5]). Of those women enrolled, 66 completed the one-year study of bone change. Sixty-two maintained menstrual cycle diary records during the study, and these are the data we report here.

For this analysis, we consider women to be either normally active nonrunners (*n* = 23) or recreational runners (*n* = 39). Recreational runners all had a stable running habit of at least two years' duration. In previous analysis we have reported data by whether, at enrolment, women expressed their intention to run a marathon during the study year. Twelve of the women who expressed this intention at enrolment actually went on to train for and complete a marathon during the one-year study.

### 2.2. Menstrual Cycle Diary and Quantitative Basal Temperature

The menstrual cycle diary includes many items which have been found to vary with the menstrual cycle and to be important for women's health and wellbeing. Individual items are ordinal variables, scored on 5-point scales. The item we denote as “fluid retention” captures women's self-reported impression of bloating, and may be indicated by a feeling of puffiness, edema, and nocturia. On the diary it is rated from 0 (none) to 4 (very intense, usually meaning edema and multiple episodes of nocturia) [15, 16]. First waking (basal) oral temperatures were measured prior to rising, and the validated [13, 14] quantitative basal temperature algorithm (QBT) was used to estimate ovulatory status (inferred from the presence or absence of a premenstrual rise in temperature), the cycle day of the basal temperature rise (which follows the LH peak by 24–36 hours, on average). Luteal phase length (the number of premenstrual days with elevated temperature following QBT rise) was used to categorize ovulatory cycles as either short luteal phase (<10 days) or normal luteal phase (≥10 days). Note that the threshold for short luteal phase by QBT is 10 days; the threshold by LH peak is 12 days.

### 2.3. Pooled Hormone Assays

Serum samples were physically pooled by mixing equal aliquots prior to radioimmunoassay for hormones including progesterone and estradiol (normal range: estradiol: 40–730 pmol/L (follicular) and 180–570 pmol/L (luteal); progesterone: 0.3–4.8 nmol/L (follicular) and 19–90 nmol/L (luteal) [4]). Serum samples were drawn during the first cycle following the two screening cycles (baseline) and at the end of the 1-year study (final). The protocol called for menstrual timing of serum samples at the mid-follicular (cycle days 3–8) and premenstrual samples. In addition to the physical pooling which prevents us from describing follicular and luteal levels separately, menstrual timing was somewhat imprecise. This was a one-year prospective study of volunteer community-dwelling women with busy lives. The screening by actual menstrual timing is described in the results section.

### 2.4. Data Summary and Statistical Analysis

Our focus was on two 11-day windows: the perimenstrual window centered on the first day of flow, and the periovulatory window, centered on the day of QBT-rise (onset of higher basal temperature). For anovulatory cycles (lacking a QBT rise) we substituted the mid-cycle window, centred on the middle cycle day, in place of a periovulatory window. To weight each woman equally, we first took an average fluid retention score for each woman across all her available menstrual cycles, before data were summarized across women and displayed graphically across each window using mean and standard error of the mean (SE). Only ovulatory cycles were included in the periovulatory window. These data were displayed overall and separately for runners and for normally active women.

For analysis of the effects of menstrual cycle timing and ovulatory status, data were summarized as average fluid retention during the periovulatory or mid-cycle window and the perimenstrual window, providing two data points per menstrual cycle. These data were analyzed with analysis of variance (ANOVA) using repeated measures of woman, and cycle-within-woman, and fixed factors of menstrual window (mid-cycle/periovulatory versus perimenstrual), ovulatory status (ovulatory versus anovulatory) and their interactions.

Finally, for each cycle for which we had hormone values, we computed an average of fluid retention over the final five (premenstrual) days. We analyzed the relationship between premenstrual fluid retention and estradiol or progesterone levels using ANOVA with sample timing (baseline or final) and hormone levels as factors. We also performed a more coarse analysis by comparing premenstrual fluid retention (present or absent) with the tertile of estradiol and progesterone levels using Fisher's exact test. Statistical analyses were performed with Stata (College Station, TX, version 9). Variability was shown as standard error of the mean (SE). Type I error rate was set at *α* = 0.05.

## 3. Results

In this observational prospective study “fluid retention” (or “bloating”) scores based on daily diary records were available from 765 cycles in 62 healthy, normal weight, initially ovulatory women ages 20–42. Four of the 66 women in the original bone density study completed all other parts of the protocol, but did not keep daily diary records, and so are excluded from this analysis.  Table 1 describes the demographic characteristics of these 62 women overall and by exercise pattern. All but two were Caucasian—one woman was Chinese and another Filipino; both of these were in the normally active group. Women in both normally active and running groups were similar in age, parity, cycle length, luteal phase length, and race. However, runners were significantly leaner with lower body mass index (BMI) values. 

Almost all (97.6%) of these 765 menstrual cycles during one year of observation were of normal length; 4 (0.5%) were shorter than 21 days and 14 (1.8%) were longer than 36 days. All women with a short cycle were nonrunners, and this was statistically significant (Fisher's exact test, *P* = 0.016), but long cycles were as likely in runners as in nonrunners (Fisher's exact test, *P* = 0.60). By study design, the two screening cycles were normally ovulatory with a sufficient luteal phase length. Among subsequent cycles during the one-year of prospective monitoring, normal ovulation occurred in 531 cycles (72%), ovulation with short luteal phase (luteal length < 10 d) in 186 cycles (25%) and anovulation (no QBT rise) in 21 cycles (2.8%). Over the course of the year, most women (*n* = 50 of 62, 81%) had one or more cycles with disturbed ovulation; 10 (16%) had one or more anovulatory cycles, and the remaining 40 (65%) ovulated consistently but with one or more short luteal phase cycles. Runners were no more likely to have ovulation disturbances than nonrunners (Fisher's exact test, *P* = 0.28).

**Figure 1:**
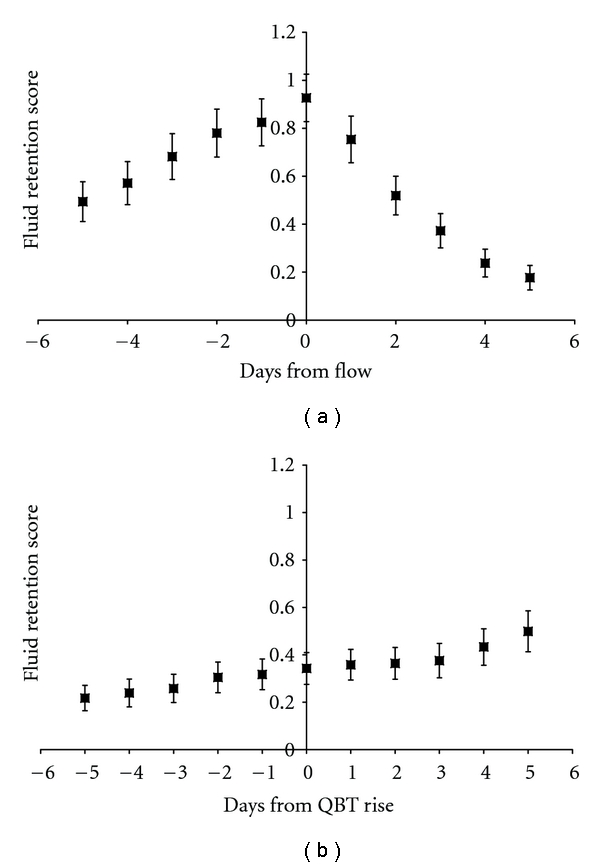
Fluid retention scores (range: 0–4) in 62 initially ovulatory women across one year by menstrual cycle day relative to (a) the onset of menstrual flow (*n* = 765, 12.3 ± 0.51 cycles per woman), where 0 is the first day of flow, and (b) in ovulatory cycles only (*n* = 717, 11.6 ± 0.54 cycles per woman), where 0 is the first day of elevated basal temperature (denoted QBT rise) as estimated by the validated quantitative basal temperature algorithm (QBT). To correct for differences in the duration of data collection by women, data are presented as the average (bars ± standard error of the mean) of within-woman averages. Fluid retention was scored from 0–4 using the daily Menstrual Cycle Diary.

Figure 1 shows the pattern of fluid retention scores across the menstrual cycle. There were 765 cycles of data (12.3 ± 0.51 cycles per woman) for the perimenstrual window (Figure 1(a)) and 717 cycles of data (11.6 ± 0.54 cycles per woman) for the periovulatory window (Figure 1(b)). Fluid retention peaked on the first day of menstruation. Raw fluid retention scores are ordinal, but averages of fluid retention scores tend towards a normal distribution (by the central limit theorem), which justifies parametric analysis of summary scores. In this healthy, nontreatment seeking population, fluid retention scores, tended to be low, and the most common score was zero. Even premenstrually (within 3 days of the onset of menstrual flow), when fluid retention was highest, few scores exceeded 1 (21% of scores within 3 days of the onset of menstruation were >1). Missing data were infrequent (<5%).

**Figure 2:**
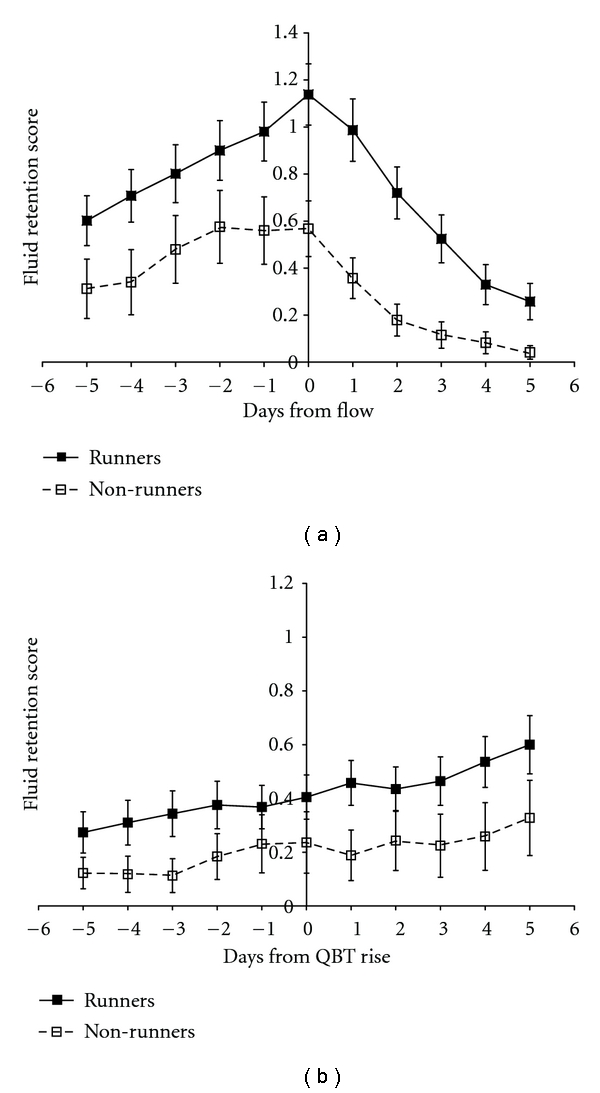
Patterns of fluid retention during the menstrual cycle for runners (*n* = 39) and normally active women (*n* = 23) relative to (a) the onset of menstrual flow, where 0 is the first day of flow, and (b) in ovulatory cycles only; the first day of elevated basal temperature is shown (denoted QBT rise) estimated by the validated quantitative basal temperature algorithm (QBT). To correct for differences in the duration of data collection by women, data are the average (bars ± standard error of the mean) of within-woman averages. Data were collected by 62 initially ovulatory women over one year. Fluid retention/“bloating” was scored from 0–4 using the Daily Menstrual Cycle diary.

By contrast with the peak in perimenstrual fluid retention, the change over the 11-day periovulatory period was a gradual rise from a score of 0.22 five days before, to a score of 0.50 five days after, the day of ovulation by QBT. Figure 2 shows the patterns in fluid retention in women who were normally active or runners. The overall temporal patterns were similar, but, contrary to our initial hypothesis, fluid retention/bloating scores for runners (*n* = 39) were consistently higher than those for normally active women (*n* = 23).

### 3.1. Fluid Retention by Cycle Timing and Ovulatory Status

**Figure 3:**
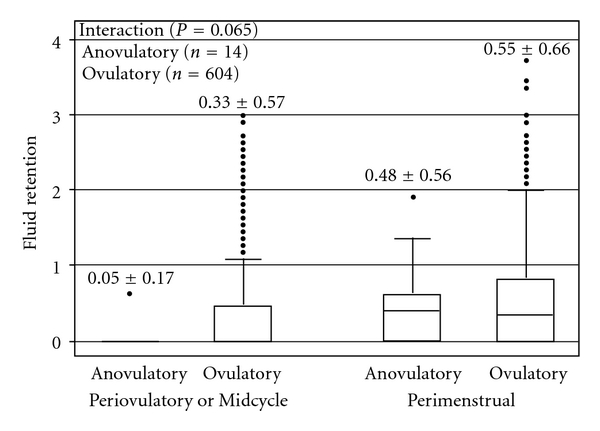
Box plots of fluid retention scores comparing data from anovulatory (*n* = 14) and ovulatory (*n* = 604) cycles averaged over the 11-day intervals of the periovulatory or mid-cycle window and the perimenstrual windows. Boxes show 25th, median, and 75th percentiles, bars show upper adjacent values, and dots represent outliers beyond that. The mean ± SE are shown above each box plot. Repeated measures analysis of variance found a nonsignificant interaction (*P* = 0.0645) between ovulatory status and menstrual timing of fluid retention, a significant effect of menstrual timing (*P* < 0.0001) and no overall effect of ovulation (*P* = 0.546). Data were collected by 62 initially ovulatory women over one year. Fluid retention/“bloating” was scored from 0–4 using daily Menstrual Cycle Diary data.

Figure 3 illustrates average fluid retention during the 11-day periovulatory/mid-cycle and perimenstrual windows for ovulatory and anovulatory cycles. Complete data were available for 618 menstrual cycles. The interaction between ovulatory status and menstrual cycle timing tended towards but did not reach statistical significance (*F* = 3.43, *P* = 0.065) by repeated measures ANOVA over women and cycles within woman. Fluid retention was significantly higher during the perimenstrual window than during the periovulatory/mid-cycle window (*F* = 32.56, *P* < 0.0001). Ovulatory and anovulatory (*n* = 14) cycles did not differ significantly (*F* = 0.36, *P* = 0.546). Only 14 of the anovulatory cycles could be included in this analysis because computing the Perimenstrual value required diary data from two consecutive cycles. There were strongly significant differences among women (*F*(61,616) = 57.42, *P* < 0.0001), but no differences among menstrual cycles within a given woman (*F*(555,616) = 1.74, *P* = 0.397).

### 3.2. Fluid Retention Relative to Estradiol and Progesterone Levels

Diary and hormone assay data were both available in 54 baseline cycles and 34 final cycles. Of the original 62 *baseline* cycles, the reasons for exclusion were wrong hormone timing (*n* = 2 early follicular) and incomplete data (*n* = 6). Of the 62 *final* cycles, the reasons for exclusion were wrong hormone timing (*n* = 5 early follicular) and incomplete data (*n* = 25). These were the samples we used for the analysis of estradiol. For progesterone analysis we further excluded samples from cycles in which QBT analysis could not be computed (*n* = 1), or where the premenstrual serum sample was drawn outside of the luteal phase (*n* = 8). Therefore the sample sizes for progesterone analysis were 47 baseline and 29 final cycles. Because of scheduling difficulties in four of the final cycles, the follicular and premenstrual blood samples were drawn from two consecutive menstrual cycles rather than within the same cycle. In these cases, the corresponding fluid retention scores were taken from the cycle in which the luteal phase blood sample was drawn; these cycles were all ovulatory by QBT. In one case, a single blood draw was taken in the luteal phase of an ovulatory cycle with no corresponding follicular phase sample. To provide comparable data with the remaining samples, we simulated dilution with a follicular sample by averaging the value with the average 2 nmol/L concentration in the follicular phase. 

In ANOVA of timing (baseline or final) and hormone levels (as continuous variables), there were no significant linear relationships between premenstrual fluid retention scores and estradiol (*F*(1,82) = 0.00, *P* = 0.97) or progesterone (*F*(1,73) = 0.47, *P* = 0.50). The data were also analyzed as categories the presence or absence of premenstrual fluid retention was also not associated with tertiles of estradiol or progesterone (Fisher's exact test *P* = 0.542 and *P* = 0.233, resp.).

## 4. Discussion

In this observational prospective study we have presented fluid retention data from 62 women who completed a menstrual diary daily for one year. This study shows that the temporal pattern of women's fluid retention experiences across the menstrual cycle is different than expected with a peak on the first day of flow rather than premenstrually. Contrary to our expectations, we also found no significant difference in fluid retention between ovulatory and anovulatory cycles (but may have been underpowered because of few anovulatory cycles). Unexpectedly, we further found higher fluid retention scores for runners (*n* = 39) than for normally active women (*n* = 23). Finally, we looked for and did not find significant relationships between premenstrual fluid retention scores and estradiol or progesterone levels. Our observation that fluid retention peaked on the first day of flow, rather than prior to flow, is in apparent contradiction to established knowledge suggesting that “bloating” is *premenstrual.* However, our observations are consistent with two other studies [2, 3]. This suggests that those who are interested in documenting menstrually changing symptoms should continue menstrual cycle record-keeping into the days following the onset of menstrual flow. 

Fluid retention scores were in a likely asymptomatic and low range in this large, one-year, prospective study of reproductively mature premenopausal women selected to be healthy, of normal weight, to have regular cycles and to be normally ovulatory. Nevertheless, a pattern of fluid retention across the menstrual cycle was apparent. The highest scores, on the first day of menstrual bleeding, averaged approximately one on a 0–4 scale. Fluid retention scores declined rapidly after the onset of menstruation and reached a nadir in the mid-follicular phase. Women's perception of puffiness or bloating then gradually increased, starting before and continuing a steady increase after ovulation. 

We did not expect to find higher fluid retention in recreational runners than in normally active women. Prospective studies from this laboratory have shown decreases in fluid retention with increasing exercise in both sedentary women [17] and in fit women who increased their training in preparation for a marathon [11]. Sedentary women just starting exercise showed the largest decreases in fluid retention. A rebound increase in fluid retention with decreasing exercise, however, has been reported in the exercise literature [18]. Although this was a prospective study, the comparison between runners and nonrunners was cross-sectional and women self-selected their activity patterns. One possible explanation for our apparently contradictory finding of greater fluid retention in runners may be that running allows women to control otherwise troublesome premenstrual symptoms including fluid retention, and so women with more symptoms may be motivated to start or to continue running. Another is that runners may have consumed more liquids because of thirst due to exercise-related fluid losses and thus had more nocturia (a component of instructions to women on scoring fluid retention). Finally, with more intense exercise, hypothalamic temperature increases and higher levels of oxytocin, vasopressin, and other neurotransmitters are released [19]. These physiological changes could lead to fluid retention that was greater in runners than in normally active women. 

Our hypothesis that we would find greater fluid retention in anovulatory cycles than ovulatory cycles was also not supported by these data. Given adequate estradiol levels to maintain regular flow, we expected that, in the absence of the antimineralocorticoid activity of progesterone [20], greater fluid retention would occur. Within-woman studies have shown greater premenstrual symptoms (one component of which is bloating or fluid retention) in those cycles with higher estradiol and lower progesterone values [21]. The interaction between ovulatory status and menstrual cycle timing did approach significance—fluid retention scores were similar in the premenstrual window between ovulatory and anovulatory cycles, but mid-cycle values (from anovulatory cycles) were lower than periovulatory ones (Figure 3). This may reflect the absence of a definite, high mid-cycle estrogen peak in anovulatory cycles. However, mean estradiol levels did not significantly differ between the anovulatory and ovulatory cycles in our published analysis of hormone data from the original study [4]. 

Although there is a clear menstrual pattern in fluid retention, the peak occurs at a time when estradiol and progesterone levels are low. Perhaps there is a lag of fluid dynamics in response to previous higher hormone levels. Fluid retention around flow was similar between anovulatory and ovulatory cycles (although we had few anovulatory cycles for this analysis); therefore self-perceived fluid retention/bloating is unlikely to be due to the actions of progesterone. Although our hormone sample size was modest, there is no indication of a relationship between pooled hormone levels of estradiol and progesterone and fluid retention scores. 

This study has several strengths. One is its relatively large size and one year's duration. Another is that all participants were carefully screened for healthy lifestyles and normal body weights prior to entry, and that all were initially ovulatory. The primary focus of the original study was on bone and exercise—women were not seeking treatment for premenstrual symptoms. Thus this study was uniquely able to describe the physiology of women's menstrual cycle-related experiences.

However, there are also several limitations to this analysis. At the time this study was designed in the early 1980s we intended to analyze all of the collected menstrual cycle diary data (which was why we collected these data when our grants provided no funding for this); however, in that era no registry required us to specify to secondary outcome variables. We have subsequently published a parallel analysis of mood symptoms using the same 62-woman strong dataset [22]. Fluid retention, commonly described by women as “bloating,” was by self-report, and the degree of fluid retention was very mild. In retrospect, we regret not collecting parallel physiological data such as waist circumference, examinations for edema, or sodium excretion rates across these cycles. These results may not generalize to women with *clinically abnormal fluid retention *or women seeking help for premenstrual symptoms. The women in this study were not typical of the general population, being leaner and more active. Heavier and less physically active women tend to have more fluid retention. Fluid retention scoring may also vary between individuals. However, the comparison of ovulatory and anovulatory cycles was within-person, so the results are robust to individual variation in scoring criteria. The hormone sampling was limited to two cycles per woman, follicular and premenstrual samples were physically pooled before assay. In addition, a proportion of the original cohort had stopped keeping the daily diary records by the final menstrual cycle of the study, which limits our ability to test specific hormonal associations. Furthermore, we excluded women with clinical androgen excess or polycystic ovary syndrome and any initial disturbances of ovulation. 

All anovulatory cycles in this sample were of normal length; we would expect a different hormonal pattern for women with oligomenorrhea from hypothalamic suppression, or for those with tonically higher estradiol levels associated with anovulatory androgen excess (polycystic ovary syndrome).

This is the first large prospective dataset documenting self-reported fluid retention as well as both ovulation and activity characteristics. This study provides new information about the patterns of perceived fluid retention across ovulatory menstrual cycles in healthy women, and documents a well-known phenomenon that many women experience. This study adds to the accumulation of knowledge regarding women's health, menstrual cycle experiences, and relationships with a natural, normal physiological process. Further research is needed to explain why maximal fluid retention occurs on the first day of flow when estradiol and progesterone levels have already decreased, and why healthy, normal weight runners should have more fluid retention than normally active women.

##  Conflict of Interests

All authors have no competing interests to declare.

## Figures and Tables

**Table 1:** Demographic and menstrual cycle information for 62 women with daily Menstrual Cycle Diary (MCD) data. Women were screened to be initially ovulatory and healthy. They participated in a one-year prospective study. The women are shown in two groups: normally active women did less than an hour of aerobic activity a week (*n* = 23) and runners include those training for a marathon plus those running for health and fitness (*n* = 39). Data are presented as mean ± SE. Statistical analyses are by *t*-test (age, height, weight, BMI, menarche, proportion of anovulatory cycles, and duration of MCD data), Fisher's exact test for contingency (parity versus exercise), and nested analysis of variance (cycle length, luteal length).

	Normally active	Runner	Statistic	*P* value	All women
	*n* = 23	*n* = 39			*n* = 62
Age	35.0 ± 1.19	33.3 ± 0.82	*t*(60) = 1.25	0.216	33.9±0.68
Height (cm)	160.9 ± 1.42	162.7 ± 0.93	*t*(60) = −1.13	0.264	162.0±0.79
Weight (kg)	59.4 ± 1.72	57.4 ± 0.80	*t*(60) = 1.21	0.230	58.1±0.81
BMI	22.9 ± 0.54	21.6 ± 0.24	*t*(60) = 2.52	0.014	22.1±0.26
Parity (%)	39% ± 10.4	36% ± 7.8	*χ*_(1)_^2^ = 0.065	1.000	37%±6.2
Race					
White	21	39			
Asian	2 (Chinese, Filipino)	0			

Cycle characteristics					
Cycle Length (d)	27.6 ± 0.18	28.4 ± 0.15	*F*(1,60) = 0.83	0.37	28.2 ± 0.12
Luteal Length* (d)	10.7 ± 0.13	10.5 ± 0.10	*F*(1,60) = 0.21	0.65	10.6 ± 0.08
% anovulatory	1.7 ± 1.09%	4.5 ± 2.16%	*t*(60) = −1.22	0.23	3.5 ± 1.42%
Days of MCD data	326.7 ± 24.77	359.5 ± 17.01			347.4 ± 14.13
Cycles of MCD data	11.8 ± 0.93	12.6 ± 0.61			12.3 ± 0.51
% missing fluid scores	4.0%	2.7%			3.2%

*Luteal lengths only for cycles that were ovulatory by QBT.
